# Evaluation of the Therapeutic Effect of Levamisole on Subclinical Mastitis in Bovine Leukemia Virus-Infected Cows Classified by Proviral Load

**DOI:** 10.3390/ani15142145

**Published:** 2025-07-21

**Authors:** Aiko Watanabe, Yosuke Maeda, Hironobu Murakami, Shiro Miyoshi, Michisaburo Miura, Koki Murao, Yasunori Shinozuka, Tomomi Kurumisawa, Kazuhiro Kawai

**Affiliations:** 1 School of Veterinary Medicine, Azabu University, Sagamihiara 252-5201, Japan; ascow19@gmail.com (A.W.); h-murakami@azabu-u.ac.jp (H.M.); y-shinozuka@ous.ac.jp (Y.S.); kurumisawa@azabu-u.ac.jp (T.K.); 2 M’s Dairy Laboratory, Chichibu 369-1412, Japan; mshiro1014@gmail.com; 3 School of Veterinary Medicine, Kitasato University, Towada 034-8628, Japan; ymaeda@vmas.kitasato-u.ac.jp; 4 Miura Dairy Clinic, Gyoda 361-0056, Japan; m_miura_014a@yahoo.co.jp; 5 Murao Animal Hospital, Ome 198-0024, Japan; murao.vet.0701@outlook.jp

**Keywords:** bovine leukemia virus, immunomodulator, levamisole, proviral load, subclinical mastitis

## Abstract

Subclinical mastitis causes economic losses due to reduced milk yield and elevated somatic cell counts (SCCs), despite no visible clinical signs. A higher incidence of subclinical mastitis has been reported in cattle infected with bovine leukemia virus (BLV). Levamisole (LMS), known for its immunomodulatory properties, has been suggested as a potential alternative to antibiotics for mastitis treatment; however, its efficacy in BLV-infected cows, particularly in relation to proviral load (PVL), remains unclear. This study aimed to evaluate the therapeutic effect of LMS on subclinical mastitis and its impact on milk immune responses by classifying BLV-infected cows based on PVL. A total of 42 dairy cows with subclinical mastitis (48 quarters) were grouped as BLV-negative, low-PVL, or high-PVL using a PVL cut-off value of 17.8 copies/10 ng DNA, and were administered LMS orally. Changes in viable bacterial counts, SCCs, and milk leukocyte populations were compared. LMS administration significantly reduced the SCC and milk macrophage numbers, especially in BLV-negative and low-PVL cows. These results suggest that LMS may improve subclinical mastitis in certain BLV-infected cows and that PVL may serve as a useful indicator for treatment responsiveness. However, the limited effect in high-PVL cows and the small sample size have limitations, warranting further investigation.

## Simple Summary

Subclinical mastitis is a common yet often overlooked condition in dairy cows, characterized by inflammation in the udder without visible clinical signs. It reduces the quality and production of milk, leading to economic losses for farmers. Cows infected with bovine leukemia virus (BLV), especially those with a high proviral load (PVL), are at increased risk of developing subclinical mastitis. This study investigated whether levamisole (LMS), an antiparasitic agent with immunomodulatory properties, could help to treat subclinical mastitis in both BLV-infected and uninfected cows. The cows were grouped based on their PVL using a cut-off value of 17.8 copies/10 ng DNA, and changes in the milk quality and immune cells were evaluated after treatment. The results showed that LMS contributed to improvements in subclinical mastitis among BLV-negative cows and those with PVL below the cut-off value. However, its effects were limited in cows with PVL above the cut-off value. These findings suggest that LMS may serve as a potential treatment option for certain BLV-infected cows with subclinical mastitis, and that the PVL cut-off value could be a useful indicator for identifying BLV-infected cows that are most likely to benefit from such treatment.

## 1. Introduction

The impact of subclinical mastitis tends to be underestimated compared with clinical mastitis, as it presents no clinical signs. However, the losses associated with subclinical mastitis on farms are significant due to increased somatic cell counts (SCCs) in milk and reduced milk yield, resulting in substantial adverse economic impacts [1]. This is widely recognized globally, and the same holds true in Japan, where increased SCCs can lead not only to reduced milk production but also to penalties and shipment restrictions based on the SCC in raw milk [2]. These factors contribute to considerable economic losses for dairy farmers.

Bovine leukemia virus (BLV) is the causative agent of enzootic bovine leukosis (EBL). The BLV infection rate in cows in Japan is high, at approximately 35% [3], and continues to spread across dairy farms. Approximately 70% of BLV-infected cows remain clinically asymptomatic and are referred to as aleukemic (AL). As the disease progresses, approximately 30% of these cows develop persistent lymphocytosis (PL), and a small percentage eventually develop EBL [4,5]. Therefore, most BLV-infected cows appear clinically healthy and are classified as either AL or PL. In recent years, it has become clear that BLV infection in cows prior to the onset of EBL can affect productivity [6,7,8]. It has also been reported that BLV-positive cows with PL have a higher incidence of subclinical mastitis compared with BLV-negative cows [9].

Following infection, BLV integrates into the animal’s genome as a provirus [10,11], and these proviral sequences remain for the host’s lifetime. The number of BLV proviral genes integrated into the host genome is referred to as the proviral load (PVL) [12,13,14], which increases as the BLV infection progresses [15]. Many recent studies have categorized PVL into high and low levels, although the criteria for these categories vary [16,17,18]. With regard to milk productivity, it has been reported that low-PVL cows do not significantly differ from BLV-negative cows in terms of the milk yield, milk fat, and milk protein at 305 days in milk, whereas high-PVL cows show reduced levels of these parameters compared with BLV-negative cows [19]. Regarding mastitis, we previously reported that high-PVL cows have lower levels of antimicrobial peptides in their milk compared with low-PVL cows and develop more severe clinical mastitis [20]. Furthermore, high-PVL cows are reportedly more susceptible to subclinical mastitis within the first 52 days in their milk compared with low-PVL and BLV-negative cows, which has led to significant economic losses in Hokkaido, Japan [21].

In Japan, the treatment of mastitis primarily relies on antibiotics [22], and dairy farmers place strong trust in their use. According to the clinical mastitis treatment flowchart, it is recommended to delay treatment in cases of mild to moderate clinical mastitis until bacterial culture tests are available, and then select the antibiotics based on the antimicrobial susceptibility testing results [22]. Other reports have indicated that many cases of mastitis with no bacterial growth recover naturally without treatment [23]. Nonetheless, dairy farmers often seek antibiotic treatment regardless of the severity of clinical signs or bacterial culture test results. The increased use of antibiotics in the livestock industry in recent years has raised concerns among public health officials. More prudent and limited antibiotic use has been advised due to concerns about food safety and the development of antimicrobial-resistant bacteria. In cases of severe clinical signs, the use of antibiotics is often unavoidable. However, for milder forms of mastitis, such as subclinical mastitis, non-antibiotic treatments have been reported, including therapies using cytokines [24] or factors such as human natural killer cells that enhance the immune system or exert antimicrobial effects [25].

Among the non-antibiotic veterinary medicines currently available in Japan for cattle, few have demonstrated immunomodulatory effects and proven efficacy against mastitis, such as reducing the SCCs or bacterial counts. Levamisole (LMS) is a broad-spectrum antiparasitic agent that also functions as an immunomodulator. It activates and normalizes weakened cellular immunity [26] and modulates the functions of T lymphocytes, macrophages, and neutrophils [27,28]. In livestock veterinary medicine, LMS has reportedly reduced SCCs and bacterial counts in dairy animals with chronic mastitis [29,30] or subclinical mastitis [31,32,33]. However, the effects of LMS administration on BLV-infected cows with mastitis have not been clarified, and no reports have examined its impact in BLV-infected cows classified according to blood PVL.

Milk macrophages in BLV-infected cows with AL or PL reportedly exhibit reduced function (phagocytic ability) [34], and the data clearly indicate that milk immunity is already suppressed before the onset of EBL. In Japan, where the number of BLV-infected cows is steadily increasing, new non-antibiotic treatments are needed to improve subclinical mastitis in immunosuppressed BLV-infected cows with AL or PL. Such treatments would not only help to reduce antibiotic use but could also serve as a countermeasure against the economic losses associated with BLV and subclinical mastitis.

In this study, we classified BLV-infected cows that developed subclinical mastitis into groups based on whether their blood PVL was above or below a cut-off value of 17.8 copies/10ng DNA [35], which we previously identified as a useful indicator of both the severity of clinical mastitis and the economic losses associated with its treatment. BLV-infected cows were treated with LMS, and its effects on subclinical mastitis and immune dynamics in milk were evaluated.

## 2. Materials and Methods

From June 2021 to October 2022, a total of 88 lactating cows (345 quarters) from 2 dairy farms in Saitama Prefecture, Japan, were screened for subclinical mastitis using the California Mastitis Test (CMT) during routine milking. Subclinical mastitis was defined as the absence of visible abnormalities in the milk or udder and no apparent systemic signs, with a SCC of 300,000 cells/mL or greater [1]. During the study period, cows with CMT-positive quarters subsequently underwent a SCC measurement. Based on these results, 42 cows (40 Holsteins and 2 Brown Swiss) with 48 affected quarters that met the criteria were enrolled in the study, with the day of diagnosis designated as Day 0. Of these, 33 cows with 39 affected quarters were randomly selected to receive LMS treatment. Information on the animals’ age, parity, and days in milk (i.e., stage of lactation) is provided in Table 1.

All cows were enrolled in the study after obtaining owner consent. This study was conducted in accordance with the regulations of the Azabu University Animal Experimentation Committee, Sagamihara, Japan (Permit No. 200708-9). Based on a previous report [35], BLV-positive cows with detectable blood PVL above the cut-off value (17.8 copies/10 ng DNA) were classified as above-PVL, whereas those with PVL below the cut-off value were classified as below-PVL. Cows with an undetectable PVL were classified as BLV-negative [5].

### 2.1. Study Design

The day of subclinical mastitis onset was defined as Day 0. After post-milking, the milk was aseptically collected from the affected quarter, and peripheral blood was collected from the tail vein using EDTA-2Na-containing vacuum blood collection tubes and Catalyst^®^ lithium heparin whole-blood separators. Subsequently, 33 cows with 39 quarters received a single oral dose of LMS hydrochloride powder·100 (Kyoritsu Seiyaku Corp., Tokyo, Japan) at 75 mg/kg. The dosage of LMS was determined based on a previous report [29]. The non-treated control group included 9 cows with 9 quarters. On Days 0, 3, 5, 7, and 14, milk was collected in the same manner, and peripheral blood was collected using EDTA-2Na-containing vacuum blood collection tubes. Milk samples for bacterial culture assessment, SCC measurement, and leukocyte population analysis were stored at 4 °C and analyzed within 24 h. The blood samples were stored at 4 °C until used for a complete blood count (CBC), blood biochemistry analyses, and BLV testing via quantitative PCR (qPCR).

### 2.2. Bacterial Culture of Milk Samples

The milk samples were aerobically cultured on 5% sheep blood agar at 37 °C for 24 h, following the procedure described by the National Mastitis Council (NMC) [36]. Microorganisms that grew on the agar plates were identified based on the colony morphology, hemolysis patterns, and Gram staining, and quantified by counting colony-forming units per mL of milk. The isolates were then stored at −80 °C and later identified to the species level following the NMC guidelines [36]. However, in cases where species-level identification was difficult or the number of isolates was small, bacterial groups were categorized at the genus or group level to ensure consistency and accuracy in the statistical analysis. The rate of change in the number of viable bacteria on Days 3, 5, 7, and 14 was investigated, with the total count on Day 0 set to 100. Contaminated milk samples from Day 0 were excluded from the viable bacterial count.

### 2.3. Measurement of SCC in Milk

The SCC was measured as previously described [37]. The cells were quantified using a DeLaval cell counter (DeLaval International AB, Tumba, Sweden).

### 2.4. Measurement of Leukocyte Population in Milk

Leukocyte populations in the milk were analyzed via staining for cell surface markers, as described in previous reports [38]. Milk fat and whey were removed by centrifugation at 700× *g* for 20 min at room temperature, and the pellet was resuspended in 4.5 mL of phosphate-buffered saline (PBS). This cell suspension was layered over 4.0 mL of Lymphosepar I (density 1.077; Immuno-Biological Laboratories, Gunma, Japan) and centrifuged at 400× *g* for 30 min at room temperature. After centrifugation, the interface was collected, washed twice with PBS, and diluted as necessary to isolate the leukocytes from the milk. The expression of leukocyte surface antigens was analyzed using monoclonal antibodies against the following cell surface markers: CD3 (MM1A, IgG1, WSU), CD4 (CACT183A, IgG1, WSU), CD8 beta (BAT82A, IgG1, WSU), gamma delta T (ILA29, IgG1, WSU), MHC-class II (CAT82A, WSU), CD14 (CAM66A, IgM, WSU), CD335 (AKS1, IgG1, AbD Serotec Inc.), CD21 (BAQ15A, IgM, WSU), and CD172a (DH59B, IgG1, WSU). The percentages of each cell type were determined by flow cytometry (FACScan; Becton Dickinson, Franklin Lakes, NJ, USA).

### 2.5. CBC Analysis

CBC parameters (red blood cells, hematocrit (Hct), hemoglobin, mean corpuscular volume, mean corpuscular hemoglobin, mean corpuscular hemoglobin concentration, platelets, white blood cells (WBCs), neutrophils, lymphocytes, monocytes, eosinophils, and basophils) in peripheral blood were determined on Days 0, 3, 5, 7, and 14 using an automated hematology analyzer (IDEXX ProCyte Dx; IDEXX Laboratories Inc., Westbrook, ME, USA) and Celltac α MEK-6558 (Nihon Kohden Corporation, Tokyo, Japan).

### 2.6. Blood Biochemistry Analysis

Blood urea nitrogen (BUN), total protein, albumin, globulin, and total cholesterol were measured on Day 0 using a blood chemistry analyzer (IDEXX Catalyst One; IDEXX Laboratories Inc., Westbrook, ME, USA).

### 2.7. Measurement of Blood PVL

PVL was measured using a previously described [39] and widely adopted method in Japan [40,41,42] for determining PVL.

#### 2.7.1. Isolation of Genomic DNA from Blood

Genomic DNA was isolated from whole-blood samples using the Wizard genomic DNA purification kit (Promega Corp., Madison, WI, USA) following the protocol provided by the manufacturer.

#### 2.7.2. Construction of Molecular Clones

The BLV proviral genome was PCR-amplified using PrimeSTAR GXL DNA polymerase (Takara Bio, Shiga, Japan), and the resulting products were self-ligated and inserted into the pSMART LC Amp vector (Lucigen, Middleton, WI, USA) using an In-Fusion HD cloning kit (Takara Bio), followed by amplification in the *Escherichia coli* strain Stbl3.

#### 2.7.3. qPCR Analysis for BLV Determination

Quantification of viral and proviral BLV genome levels was performed for all samples by qPCR using the 7500 Real-Time PCR system (Applied Biosystems, Foster City, USA). The primers (5′-GGACAAATGGACTGCTCAAAC-3′ and 5′-CTCCCATCTGGTCTTTAGAATTG-3′) and probe (5′-FAM-CTTCCCATGACTCAGGCCCTTTCT-TAM-3′) used in this study were designed to target a highly conserved region of the BLV polymerase gene for the detection of PVL. A standard curve was generated from serial dilutions of pBLV-FLK plasmid, which was constructed as previously described [39] (GenBank accession number: LC164083), and was used to calculate the proviral copy number.

### 2.8. Statistical Analysis

Comparisons were made between the following groups:

1. Above-PVL groups: LMS-treated vs. LMS-untreated;
2. LMS-treated groups: BLV-positive vs. BLV-negative;
3. LMS-treated, BLV-positive groups: Above-PVL vs. Below-PVL.

Here, Above-PVL and Below-PVL refer to cows with PVL above and below the cut-off value, respectively, as previously reported [35]. 

To compare the pathogen factors on the day of subclinical mastitis onset (Day 0) for comparisons (1) through (3), Fisher’s exact test was used. The Wilcoxon rank-sum test was used to compare host factors (age, parity, and days in milk) for comparisons (1) through (3) as well as the blood biochemistry parameters, CBC, SCC, and leukocyte populations in milk on Day 0. The Wilcoxon signed-rank test was used to evaluate the effect of LMS administration by comparing the CBC, SCC, and leukocyte populations in milk among all groups on Days 3, 5, 7, and 14 (relative to Day 0), as well as changes in the blood PVL between Days 0 and 14.

All statistical analyses were performed using the IBM SPSS Statistics 27.0 software (International Business Machines Co., Ltd., Armonk, NY, USA). A *p*-value of less than 0.05 was considered statistically significant.

## 3. Results

### 3.1. Cows

Of the 48 quarters from 42 cows included in this investigation, 30 cows (32 quarters) were BLV-positive with PVL in the blood detected by qPCR, and 12 cows (16 quarters) were BLV-negative, with no PVL detected in the blood. The BLV-positive, LMS-treated group consisted of 21 cows with 23 quarters (Above-PVL: 17 quarters from 15 cows; Below-PVL: 6 quarters from 6 cows), whereas the BLV-negative group included 16 quarters from 12 cows. The BLV-positive, LMS-untreated group included 9 quarters of 9 cows (all Above-PVL).

### 3.2. Comparison of Host, Pathogen Factors, CBC, and Blood Biochemistry Parameters (Day 0)

No significant differences in the host or pathogen factors were observed (Table 1 and Table 2).

Blood biochemistry analysis: BUN was significantly lower in the LMS-treated BLV-negative group compared with the LMS-treated BLV-positive group (*p* = 0.005) (Table S1).

CBC: Compared to the LMS-untreated group above the PVL cut-off value, the LMS-treated group above the PVL cut-off value had significantly lower hemoglobin (*p* = 0.048) and Hct (*p* = 0.035) levels, and a significantly higher basophil count (*p* = 0.015) (Table S1).

### 3.3. Dynamics of PVL and Trends in CBC in Peripheral Blood

**Figure 1:**
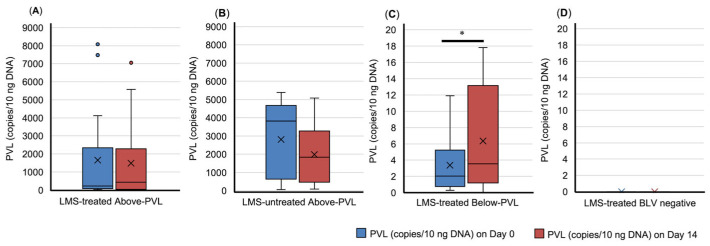
Dynamics of bovine leukemia virus provirus load (PVL) in blood. (**A**) LMS-treated Above-PVL group; (**B**) LMS-untreated Above-PVL group; (**C**) LMS-treated Below-PVL groups; (**D**) LMS-treated BLV-negative group. Blue box: PVL (copies/10 ng DNA) on Day 0. Red box: PVL (copies/10 ng DNA) on Day 14. Abbreviation: LMS, levamisole. Above-PVL: group with PVL above the cut-off value; Below-PVL: group with PVL below the cut-off value. * *p* < 0.05. The cross sign indicates the mean. The blue and red dots represent outliers.

In the LMS-treated group below the PVL cut-off value, the blood PVL on Day 14 was significantly higher than on Day 0 (*p* = 0.046) (Figure 1). In the LMS-treated BLV-negative group, no seroconversion was observed during this study (Figure 1).

In the LMS-treated BLV-negative group, the WBC on Day 3 was significantly higher on Day 0 (*p* = 0.049) (Figure S1). Additionally, the monocyte count was significantly higher on Day 5 in the LMS-untreated group above the PVL cut-off value (*p* = 0.015) and on Day 7 in the LMS-treated group above the PVL cut-off value (*p* = 0.046), LMS-treated BLV-positive group (*p* = 0.042), and LMS-treated BLV-negative group (*p* = 0.021) compared with Day 0 (Figure S1). No significant differences were observed in the other parameters (Figure S1).

### 3.4. Rate of Change in the Number of Viable Bacteria

**Figure 2:**
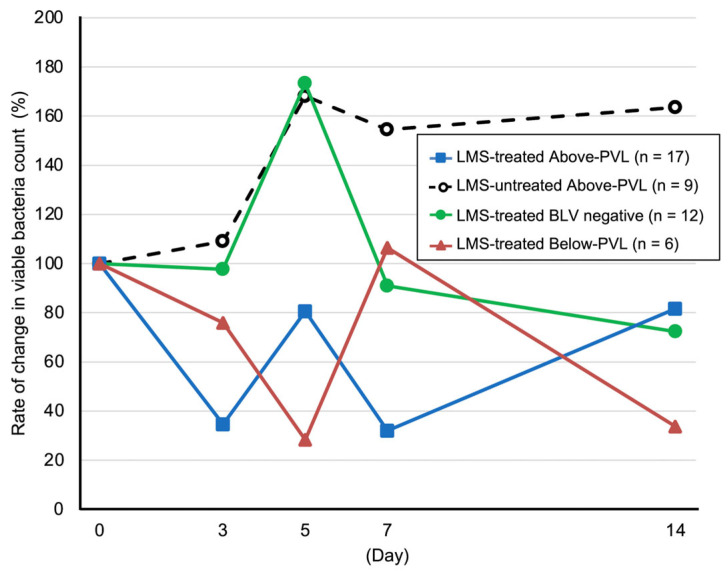
Rate of change in viable bacteria count. Blue line with squares: LMS-treated Above-PVL group (*n* = 17). Black dotted line with white circles: LMS-untreated Above-PVL group (*n* = 9). Green line with circles: LMS-treated BLV-negative group (*n* = 12). Red line with triangles: LMS-treated Below-PVL group (*n* = 6). The rate of change in the number of viable bacteria on Days 3, 5, 7, and 14 was calculated relative to the total count on Day 0, which was set to 100. Abbreviations: LMS, levamisole; PVL, provirus load; BLV, Bovine leukemia virus. Above-PVL: group with PVL above the cut-off value; Below-PVL: group with PVL below the cut-off value.

Compared with Day 0, the LMS-treated group above the PVL cut-off value showed a reduction in the viable bacterial count on Days 3, 5, 7, and 14 (Figure 2). By contrast, the LMS-untreated group above the PVL cut-off value showed increases in the viable bacterial count on all these days (Figure 2). In the LMS-treated BLV-negative group, the viable bacterial count transiently increased on Day 5; in the LMS-treated group below the PVL cut-off value, the viable bacterial count transiently increased on Day 7. However, this count decreased on the other days (Figure 2).

### 3.5. Trends in SCC and Leukocyte Populations in Milk

#### 3.5.1. SCC

**Figure 3:**
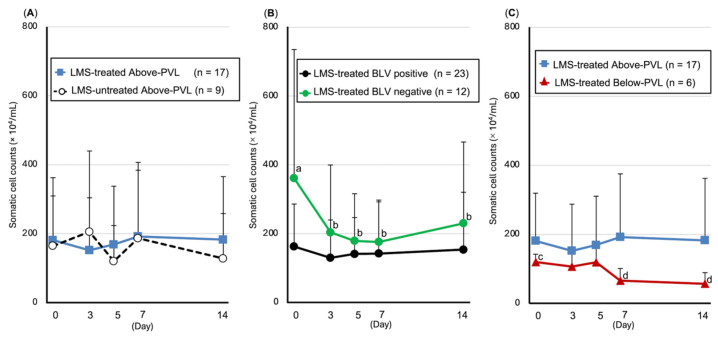
Trends in somatic cell count (SCC, mean ± SD). (**A**) Above-PVL groups: LMS-treated vs. LMS-untreated; (**B**) LMS-treated groups: BLV-positive vs. BLV-negative; (**C**) LMS-treated, BLV-positive groups: Above-PVL vs. Below-PVL. Blue box: PVL (copies/10 ng DNA) on Day 0. Blue line with squares: LMS-treated Above-PVL group (*n* = 17); Black dotted line with white circles: LMS-untreated Above-PVL group (*n* = 9); Black line with circles: LMS-treated BLV-positive group (*n* = 23); Green line with circles: LMS-treated BLV-negative group (*n* = 12); Red line with triangles: LMS-treated Below-PVL group (*n* = 6). Abbreviations: LMS, levamisole; PVL, provirus load; BLV, Bovine leukemia virus. Above-PVL: group with PVL above the cut-off value. Below-PVL: group with PVL below the cut-off value. (a–b, c–d: *p* < 0.05).

Compared with pre-LMS administration, a significant decrease in the SCC was observed in the BLV-negative group on Days 3 (*p* = 0.02), 5 (*p* = 0.015), 7 (*p* = 0.013), and 14 (*p* = 0.011), and a significant decrease in the SCC was observed on Days 7 (*p* = 0.028) and 14 (*p* = 0.028) in the group with PVL below the cut-off value (Figure 3).

#### 3.5.2. Leukocyte Populations in Milk

**Figure 4:**
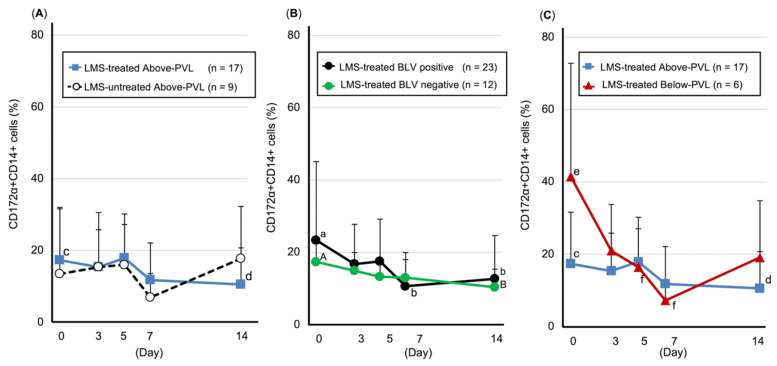
Trends in the number of CD172a-positive/CD14-positive cells (monocytes/macrophages, mean ± SD). (**A**) Above-PVL groups: LMS-treated vs. LMS-untreated. (**B**) LMS-treated groups: BLV-positive vs. BLV-negative. (**C**) LMS-treated, BLV-positive groups: Above-PVL vs. Below-PVL. Blue line with squares: LMS-treated Above-PVL group (*n* = 17); Black dotted line with white circles: LMS-untreated Above-PVL group (*n* = 9); Black line with circles: LMS-treated BLV-positive group (*n* = 23); Green line with circles: LMS-treated BLV-negative group (*n* = 12); Red line with triangles: LMS-treated Below-PVL group (*n* = 6). Abbreviations: LMS, levamisole; PVL, provirus load; BLV, Bovine leukemia virus. Above-PVL: group with PVL above the cut-off value. Below-PVL: group with PVL below the cut-off value (a–b, c–d, e–f: *p* < 0.05; A–B: *p* < 0.1).

Number of CD172a-positive/CD14-positive cells (monocytes/macrophages): Compared with pre-LMS administration, the LMS-treated BLV-positive group showed a significant decrease on Days 7 (*p* = 0.043) and 14 (*p* = 0.004), whereas the LMS-treated group above the PVL cut-off value showed a significant decrease on Day 14 (*p* = 0.013). The LMS-treated group below the PVL cut-off value showed a significant decrease on Days 5 (*p* = 0.046) and 7 (*p* = 0.046) (Figure 4). Furthermore, the BLV-negative, LMS-treated group showed a decreasing trend on Day 14 (*p* = 0.07) (Figure 4).

Number of CD21-positive cells (B lymphocytes): In the LMS-treated BLV-negative group, a significant decrease was observed on Days 7 (*p* = 0.033) and 14 (*p* = 0.039) compared with the pre-LMS administration, and the percentages were significantly lower compared with the LMS-treated BLV-positive group on Days 7 (*p* = 0.035) and 14 (*p* = 0.024) (Figure S2). In the LMS-treated below the PVL cut-off value group, a significant decrease was observed on Day 3 (*p* = 0.043) compared with the pre-LMS administration (Figure S2).

Number of CD335-positive cells (natural killer cells): Compared with the pre-LMS administration, the LMS-treated BLV-negative group showed a significant decrease on Day 14 (*p* = 0.011) (Figure S3).

Number of WC1-positive cells (gamma delta T lymphocytes): Compared with the pre-LMS administration, the LMS-treated BLV-negative group showed a significant decrease on Day 14 (*p* = 0.014) (Figure S4).

No significant differences were observed for the other leukocyte populations (Figures S5–S8).

## 4. Discussion

In this study, LMS administration resulted in reductions in viable bacterial count and SCC in cows with subclinical mastitis, specifically, BLV-negative cows and BLV-positive cows with a PVL below 17.8 copies/10 ng DNA. These findings suggest that LMS treatment ameliorates subclinical mastitis in both BLV-negative and BLV-positive cows with a PVL value below the cut-off of 17.8 copies/10 ng DNA.

Several products other than LMS have been identified that exhibit immunomodulatory effects and efficacy against mastitis when administered to dairy cows [24,25]; however, these are not generally available to dairy farmers. In addition to LMS, currently available products shown to have immunomodulatory effects and efficacy against mastitis include fat-soluble vitamins such as vitamins A and E [43,44,45], and water-soluble vitamins such as biotin [46,47]. All dairy cows examined in the present study were confirmed to have been sufficiently supplemented with these vitamins through the feed formulation prior to inclusion, indicating that the observed effects of LMS were unlikely to have been influenced by vitamin supplementation.

LMS is an anthelmintic drug that also exhibits immunomodulatory effects [26,27,28]. Research exploring this property for the prevention and treatment of mastitis has been ongoing for many years. LMS has been reported to reduce SCCs and bacterial counts in both chronic mastitis [29,30] and subclinical mastitis [31,32,33], and has demonstrated effectiveness in preventing mastitis after calving [48,49]. A report summarizing the application of LMS for mastitis treatment [50] noted that while some cows do not respond to LMS and its effect may be transient, the effectiveness of LMS against chronic mastitis has been confirmed through clinical and basic research, including immunological support. The present findings are consistent with previous reports showing that LMS reduces SCCs and bacterial counts. Moreover, this study extends earlier work by stratifying cows according to their BLV infection status and PVL levels, and by evaluating the effects of LMS within each group.

Comparison (1): In the comparison between LMS-treated groups with PVL above and below the cut-off, both groups showed a reduction in viable bacterial count and in the number of CD172a-positive/CD14-positive cells (monocytes/macrophages) in their milk. However, the SCC decreased significantly only in the group with PVL below the cut-off. This suggests that the LMS treatment may still be effective even if the PVL exceeds 17.8 copies/10 ng DNA.

Comparison (2): In the comparison between LMS-treated BLV-positive cows and LMS-treated BLV-negative cows, the viable bacterial count decreased in both groups, whereas the SCC decreased only in the BLV-negative group. The number of CD172a-positive/CD14-positive cells (monocytes/macrophages) in milk decreased in the BLV-positive group and showed a decreasing trend in the BLV-negative group. LMS reportedly enhances the phagocytic function of macrophages [28]. Although the exact mechanism remains unclear, some studies have linked this enhancement to increased cyclic guanosine monophosphate levels in macrophages [51]. Another study has suggested that LMS strongly induces the release of intracellular calcium ions by mononuclear phagocytes [52], which may contribute to this effect. Macrophages are known to play a crucial role not only in phagocytosing invading bacteria but also in rapidly engulfing neutrophils that have fulfilled their role, thereby minimizing inflammation-related damage to mammary epithelial tissue [53]. Furthermore, the phagocytic activity of milk macrophages in BLV-infected cows has been reported to be lower than in BLV-negative cows, regardless of the BLV pathology [34]. As the SCC decreased in the LMS-treated BLV-negative group, we hypothesize that LMS administration enhanced the function of macrophages in milk, promoting antigen elimination and a more rapid resolution of the intramammary infection compared with the LMS-treated BLV-positive group. However, in this study, we did not obtain data regarding the functional status of macrophages in milk, and so, detailed insights into their phagocytic ability are limited. Further investigations into macrophage function are needed.

Comparison (3): In the comparison between the LMS-treated groups with PVL above and below the cut-off value, both groups showed a reduction in the viable bacterial count and the number of CD172a-positive/CD14-positive cells (monocytes/macrophages) in milk. However, a significant decrease in the SCC was observed only in the group with PVL below the cut-off value. Among the BLV-positive cows, those with a higher PVL have been reported to express lower levels of the gene encoding IFN-gamma, the most potent activator of macrophages, than cows with low PVL [54]. These data suggest that LMS administration may have contributed to macrophage activation even in the group above the cut-off value, although the response might have been weaker when compared with the group with PVL below the cut-off value. As a result, intramammary infection may not have been sufficiently resolved, which could explain the absence of a significant reduction in SCCs in the group above the PVL cut-off value. However, further studies are needed to clarify the functional capacity of macrophages at varying PVL levels.

Previous studies on LMS-based treatment of subclinical mastitis observed reductions in the bacterial count on the third [33] and fourth [32] days after treatment. In the present study, it was presumed that LMS administration enhanced the phagocytic activity of macrophages in milk, leading to reduced bacterial counts in both LMS-treated BLV-positive groups (above and below the PVL cut-off value) and the LMS-treated BLV-negative group.

Among the housed cattle in Japan, 70–85% are reportedly infected with nematodes [55]. Although species-specific differences exist, studies in ewes have found associations between gastrointestinal nematode infections and an increased risk of subclinical mastitis, as well as elevated SCCs, when compared to non-infected ewes [56,57]. Furthermore, administration of anthelmintics in ewes reportedly led to a reduction in the SCCs [58]. These reports support the possibility that the anthelmintic activity of LMS may contribute to the improvement of mastitis. However, in the present study, SCCs decreased only in the LMS-treated BLV-negative group and the group with PVL below the cut-off value, suggesting, as previously described, that rapid infection resolution may have resulted from LMS’s direct effects on macrophages. However, as we did not confirm whether the dairy cows examined in this study were infected with nematodes—which are the target in LMS treatment—the relationship between LMS administration and the observed SCC reduction in these groups should be interpreted with caution.

Other studies have reported that LMS enhances the differentiation rate of T lymphocytes [26] and increases the number of B lymphocytes in milk from healthy cows [59]. It has also been reported that cows with mastitis have fewer T and B lymphocytes in their milk when compared to healthy cows [59]. Although an increase in T lymphocytes in peripheral blood and milk was expected following LMS administration, no such increase was observed. It has also been reported that if an animal is unable to produce the LMS-induced serum factors necessary for enhancing lymphocyte function, it will not respond clinically, even with proper LMS administration [60]. This suggests that the lack of change in T lymphocyte numbers observed in this study may be due to some cows being unable to produce LMS-induced serum factors.

In cows with PVL below the cut-off value of 17.8 copies/10 ng DNA, the blood PVL increased within the low range (still below 17.8 copies/10 ng DNA) 14 days after LMS administration. Previous studies have reported that LMS administration does not affect the antibody titers or viral replication in BLV-infected cattle [61]. As the PVL increased across a very low range, further long-term follow-up studies are needed to assess the impact of LMS on PVL dynamics.

The results of this study suggest that LMS administration may enhance the function of macrophages in milk, contributing to reductions in viable bacterial counts and SCCs. This indicates that LMS ameliorates subclinical mastitis in BLV-negative cows and BLV-infected cows with PVL below the cut-off value of 17.8 copies/10 ng DNA. Furthermore, LMS administration reduced the viable bacterial count even in cows with PVL above the cut-off value of 17.8 copies/10 ng DNA, suggesting that LMS treatment may still be effective in this group. The PVL cut-off value of 17.8 copies/10 ng DNA may be a useful criterion for evaluating the effectiveness of immunomodulatory agents in improving subclinical mastitis in BLV-infected cows. PVL has been widely used as a marker of disease progression and immunosuppression in BLV-infected cattle, with high PVL levels associated with reduced immune function [54]. In this study, stratifying cows by PVL allowed us to identify the differential responses to LMS, supporting the utility of PVL as an indicator of treatment responsiveness in BLV-infected cows. The findings of this study may offer practical insights into the application of LMS in mastitis management practices, particularly as alternatives to antibiotics are increasingly sought. Moreover, in Japan, where BLV infections are increasing, these results may also guide approaches to improving mastitis in BLV-infected cows. However, the functional activities of immune cells in milk following LMS administration remain unclear, and the small sample size and limited statistical power of this study represent limitations. Further studies involving a larger number of animal samples are thus necessary in order to more thoroughly investigate the effects of LMS on subclinical mastitis in BLV-infected cows.

## 5. Conclusions

LMS administration resulted in reduced bacterial counts and SCCs in BLV-negative cows and in BLV-infected cows with PVL below 17.8 copies/10 ng DNA. These effects suggest that LMS may enhance immune clearance within the mammary gland and contribute to the improvement of subclinical mastitis. A decrease in CD172a-positive/CD14-positive cells was observed in these groups, supporting a potential immunomodulatory role. In contrast, cows with high PVL showed a bacterial reduction without SCC improvement, indicating a limited response. The PVL cut-off value may serve as a useful indicator for identifying animals most likely to benefit from LMS treatment. However, the therapeutic effect in high-PVL cows was limited, suggesting a PVL-dependent response. Further studies are warranted to explore the underlying immunological mechanisms and assess the potential role of parasitic co-infections.

## Figures and Tables

**Table 1:** Comparison of host and pathogenic factors (Day 0): (1) Above-PVL groups: LMS-treated vs. LMS-untreated; (3) LMS-treated, BLV-positive groups: Above-PVL vs. Below-PVL.

	LMS-Treated		LMS-Untreated		
	Above-PVL	Below-PVL	Above-PVL	*p*-Value	
Variables	17 quarters, 15 cows	6 quarters, 6 cows	9 quarters, 9 cows	(1)	(3)
Age (year)	5.5 ± 1.7	4.3 ± 1.3	4.7 ± 1.3	0.347	0.154
Parity	3.3 ± 1.5	2.0 ± 0.9	2.4 ± 1.5	0.138	0.055
Days in Milk	248.3 ± 93.6	308.8 ± 69.7	252.2 ± 141.7	0.953	0.267
Pathogen					
*Streptococcus* spp.	2	2	0		
*Enterococcus* spp.	2	3	0		
*Coagulase-negative Staphylococcus* spp.	2	0	2		
*Coliform bacteria*	0	0	1	0.315	0.224
*Corynebacterium bovis*	1	0	1		
*Staphylococcus aureus*	0	0	0		
No Growth	9	1	4		
Unknown	1	0	1		

Abbreviations: LMS, levamisole; BLV, bovine leukemia virus; PVL, provirus load. Above-PVL: group with PVL above the cut-off value; Below-PVL: group with PVL below the cut-off value. Age (year), Parity, and Days in Milk are presented as mean ± SD. No growth: No bacterial growth observed on culture of milk samples.

**Table 2:** Comparison of host and pathogenic factors (Day 0): (2) LMS-treated groups: BLV-positive vs. BLV-negative.

	LMS-Treated		
	BLV-Positive	BLV-Negative	*p*-Value
Variables	23 quarters, 21 cows	16 quarters, 12 cows	(2)
Age (year)	5.2 ± 1.7	5.1 ± 2.1	0.699
Parity	2.9 ± 1.4	2.9 ± 1.4	0.868
Days in Milk	265.6 ± 90.2	229.0 ± 89.5	0.258
Pathogen			
*Streptococcus* spp.	2	2	
*Enterococcus* spp.	5	5	
*Coagulase-negative Staphylococcus* spp.	2	2	
*Coliform bacteria*	0	0	0.780
*Corynebacterium bovis*	1	0	
*Staphylococcus aureus*	0	2	
No Growth	10	7	
Unknown	1	0	

Abbreviations: LMS, levamisole; BLV, bovine leukemia virus. Age (year), Parity, and Days in Milk are presented as mean ± SD. No growth: No bacterial growth observed on the culture of milk samples.

## Data Availability

The data presented in this study are available in this article (and Supplementary Materials).

## Supplementary Materials

The following supporting information can be downloaded from https://www.mdpi.com/article/10.3390/ani15142145/s1—Table S1: Comparison of complete blood count (CBC) and blood biochemistry analyses parameters (Day 0); Figure S1: Trends in complete blood count (CBC) parameters in peripheral blood; Figure S2: Trends in the number of CD21-positive cells (B lymphocytes); Figure S3: Trends in the number of CD335-positive cells (natural killer cells); Figure S4: Trends in the number of WC1-positive cells (gamma delta T lymphocytes); Figure S5: Trends in the number of CD172a-positive/CD14-negative cells (granulocytes); Figure S6: Trends in the number of CD3 positive cells (total T lymphocytes); Figure S7: Trends in the number of CD4 positive cells (helper T lymphocytes); Figure S8: Trends in the number of CD8 positive cells (killer T lymphocytes).

## Abbreviations

SCC	Somatic cell counts
BLV	Bovine leukemia virus
EBL	Enzootic bovine leukosis
AL	Aleukemic
PL	Persistent lymphocytosis
PVL	Proviral load
LMS	Levamisole
CMT	California Mastitis Test
CBC	Complete blood count
qPCR	Quantitative PCR
NMC	National Mastitis Council
PBS	Phosphate-buffered saline
Hct	Hematocrit
WBCs	White blood cells
BUN	Blood urea nitrogen
