# Supplementary material for: Evaluation of the Therapeutic Effect of Levamisole on Subclinical Mastitis in Bovine Leukemia Virus-Infected Cows Classified by Proviral Load
Source: Animals (Basel). 2025 Jul 21;15(14):2145. doi: 10.3390/ani15142145 (PMC12291948; doi:10.3390/ani15142145)
Supplement: Supplementary file 1 [file animals-15-02145-s001.zip › Supplementary Files/Supplementary Figure captions.docx]

**Supplementary Figure S1.** Trends in complete blood count (CBC) parameters in peripheral blood (mean ± SD). (**A**) White blood cells (WBC); (**B**) Neutrophils; (**C**) Lymphocytes; (**D**) Monocytes. **Blue line with squares:**LMS-treated Above-PVL group (n = 15); **Black dotted line with white circles**: LMS-untreated Above-PVL group (n = 9); **Black line with circles**: LMS-treated BLV-positive group (n = 21); **Green line with circles**: LMS-treated BLV-negative group (n = 12); **Red line with triangles**: LMS-treated Below-PVL group (n = 6). Abbreviations: LMS, levamisole; PVL, provirus load; BLV, Bovine leukemia virus. Above-PVL: group with PVL above the cut-off value. Below-PVL: group with PVL below the cut-off value. (a-b: *P* < 0.05).

**Supplementary Figure S2.** Trends in the Number of CD21-positive cells (B lymphocytes, mean ± SD). (**A**) Above-PVL groups: LMS-treated vs. LMS-untreated; (**B**) LMS-treated groups: BLV-positive vs. BLV-negative; (**C**) LMS-treated, BLV-positive groups: Above-PVL vs. Below-PVL. **Blue line with squares:**LMS-treated Above-PVL group (n = 17); **Black dotted line with white circles**: LMS-untreated Above-PVL group (n = 9); **Black line with circles**: LMS-treated BLV-positive group (n = 23); **Green line with circles**: LMS-treated BLV-negative group (n = 12); **Red line with triangles**: LMS-treated Below-PVL group (n = 6). Abbreviations: LMS, levamisole; PVL, provirus load; BLV, Bovine leukemia virus. Above-PVL: group with PVL above the cut-off value. Below-PVL: group with PVL below the cut-off value. (a-b, c-d, *: *P* < 0.05).

**Supplementary Figure S3.** Trends in the Number of CD335-positive cells (natural killer cells, mean ± SD). (**A**) Above-PVL groups: LMS-treated vs. LMS-untreated; (**B**) LMS-treated groups: BLV-positive vs. BLV-negative; (**C**) LMS-treated, BLV-positive groups: Above-PVL vs. Below-PVL. **Blue line with squares:**LMS-treated Above-PVL group (n = 17); **Black dotted line with white circles**: LMS-untreated Above-PVL group (n = 9); **Black line with circles**: LMS-treated BLV-positive group (n = 23); **Green line with circles**: LMS-treated BLV-negative group (n = 12); **Red line with triangles**: LMS-treated Below-PVL group (n = 6). Abbreviations: LMS, levamisole; PVL, provirus load; BLV, Bovine leukemia virus. Above-PVL: group with PVL above the cut-off value. Below-PVL: group with PVL below the cut-off value. (a-b: *P* < 0.05).

**Supplementary Figure S4.** Trends in the Number of WC1-positive cells (gamma delta T lymphocytes, mean ± SD). (**A**) Above-PVL groups: LMS-treated vs. LMS-untreated; (**B**) LMS-treated groups: BLV-positive vs. BLV-negative; (**C**) LMS-treated, BLV-positive groups: Above-PVL vs. Below-PVL. **Blue line with squares:**LMS-treated Above-PVL group (n = 17); **Black dotted line with white circles**: LMS-untreated Above-PVL group (n = 9); **Black line with circles**: LMS-treated BLV-positive group (n = 23); **Green line with circles**: LMS-treated BLV-negative group (n = 12); **Red line with triangles**: LMS-treated Below-PVL group (n = 6). Abbreviations: LMS, levamisole; PVL, provirus load; BLV, Bovine leukemia virus. Above-PVL: group with PVL above the cut-off value. Below-PVL: group with PVL below the cut-off value. (a-b: *P* < 0.05).

**Supplementary Figure S5.** Trends in the Number of CD172a-positive/CD14-negative cells (granulocytes, mean ± SD). (**A**) Above-PVL groups: LMS-treated vs. LMS-untreated; (**B**) LMS-treated groups: BLV-positive vs. BLV-negative; (**C**) LMS-treated, BLV-positive groups: Above-PVL vs. Below-PVL. **Blue line with squares:**LMS-treated Above-PVL group (n = 17); **Black dotted line with white circles**: LMS-untreated Above-PVL group (n = 9); **Black line with circles**: LMS-treated BLV-positive group (n = 23); **Green line with circles**: LMS-treated BLV-negative group (n = 12); **Red line with triangles**: LMS-treated Below-PVL group (n = 6). Abbreviations: LMS, levamisole; PVL, provirus load; BLV, Bovine leukemia virus. Above-PVL: group with PVL above the cut-off value. Below-PVL: group with PVL below the cut-off value.

**Supplementary Figure S6.** Trends in the Number of CD3 positive cells (total T lymphocytes, mean ± SD). (**A**) Above-PVL groups: LMS-treated vs. LMS-untreated; (**B**) LMS-treated groups: BLV-positive vs. BLV-negative; (**C**) LMS-treated, BLV-positive groups: Above-PVL vs. Below-PVL. **Blue line with squares:**LMS-treated Above-PVL group (n = 17); **Black dotted line with white circles**: LMS-untreated Above-PVL group (n = 9); **Black line with circles**: LMS-treated BLV-positive group (n = 23); **Green line with circles**: LMS-treated BLV-negative group (n = 12); **Red line with triangles**: LMS-treated Below-PVL group (n = 6). Abbreviations: LMS, levamisole; PVL, provirus load; BLV, Bovine leukemia virus. Above-PVL: group with PVL above the cut-off value. Below-PVL: group with PVL below the cut-off value.

**Supplementary Figure S7.** Trends in the Number of CD4 positive cells (helper T lymphocytes, mean ± SD). (**A**) Above-PVL groups: LMS-treated vs. LMS-untreated; (**B**) LMS-treated groups: BLV-positive vs. BLV-negative; (**C**) LMS-treated, BLV-positive groups: Above-PVL vs. Below-PVL. **Blue line with squares:**LMS-treated Above-PVL group (n = 17); **Black dotted line with white circles**: LMS-untreated Above-PVL group (n = 9); **Black line with circles**: LMS-treated BLV-positive group (n = 23); **Green line with circles**: LMS-treated BLV-negative group (n = 12); **Red line with triangles**: LMS-treated Below-PVL group (n = 6). Abbreviations: LMS, levamisole; PVL, provirus load; BLV, Bovine leukemia virus. Above-PVL: group with PVL above the cut-off value. Below-PVL: group with PVL below the cut-off value.

**Supplementary Figure S8.** Trends in the Number of CD8 positive cells (killer T lymphocytes, mean ± SD). (**A**) Above-PVL groups: LMS-treated vs. LMS-untreated; (**B**) LMS-treated groups: BLV-positive vs. BLV-negative; (**C**) LMS-treated, BLV-positive groups: Above-PVL vs. Below-PVL. **Blue line with squares:**LMS-treated Above-PVL group (n = 17); **Black dotted line with white circles**: LMS-untreated Above-PVL group (n = 9); **Black line with circles**: LMS-treated BLV-positive group (n = 23); **Green line with circles**: LMS-treated BLV-negative group (n = 12); **Red line with triangles**: LMS-treated Below-PVL group (n = 6). Abbreviations: LMS, levamisole; PVL, provirus load; BLV, Bovine leukemia virus. Above-PVL: group with PVL above the cut-off value. Below-PVL: group with PVL below the cut-off value.
