# Supplementary material for: Evaluation of the Therapeutic Effect of Levamisole on Subclinical Mastitis in Bovine Leukemia Virus-Infected Cows Classified by Proviral Load
Source: Animals (Basel). 2025 Jul 21;15(14):2145. doi: 10.3390/ani15142145 (PMC12291948; doi:10.3390/ani15142145)
Supplement: Supplementary file 1 [file animals-15-02145-s001.zip › Supplementary Files/Supplementary Table S1.docx]

**Supplementary Table S1.** Comparison of Complete blood count (CBC) and blood biochemistry analyses parameters (Day 0).

|  | (1) Above-PVL | |  | (2) LMS-treated | |  | (3) LMS-treated | |  |
| --- | --- | --- | --- | --- | --- | --- | --- | --- | --- |
|  | **LMS-treated** | **LMS-untreated** |  | **BLV positive** | **BLV negative** |  | **Above-PVL** | **Below-PVL** |  |
| **Parameters** | (17 quarters  15 cows) | (9 quarters  9 cows) | ***P*- value** | (23 quarters  21 cows) | (16 quarters  12 cows) | ***P*- value** | (17 quarters  15 cows) | (6 quarters  6 cows) | ***P*- value** |
| BUN (mg/dL) | 8.1 ± 2.8 | 10.8 ± 5.2 | 0.446 | 8.8 ± 2.7 | 5.5 ± 2.5 | 0.005^*^ | 8.1 ± 2.8 | 10.5 ± 1.8 | 0.132 |
| TP (g/dL) | 8.9 ± 0.8 | 8.7 ± 0.4 | 0.482 | 9.2 ± 1.0 | 9.6 ± 1.1 | 0.385 | 8.9 ± 0.8 | 9.8 ± 1.2 | 0.006 |
| ALB (g/dL) | 3.2 ± 0.4 | 3.2 ± 0.2 | 1.000 | 3.2 ± 0.4 | 3.3 ± 0.7 | 0.175 | 3.2 ± 0.4 | 3.3 ± 0.3 | 0.97 |
| GLOB (g/dL) | 5.7 ± 0.9 | 5.5 ± 0.4 | 0.482 | 5.9 ± 1.1 | 6.4 ± 1.1 | 0.291 | 5.7 ± 0.9 | 6.6 ± 1.1 | 0.08 |
| T-chol (mg/dL) | 177.0 ± 43.7 | 198.8 ± 40.8 | 0.318 | 181.8 ± 40.4 | 176.4 ± 30.9 | 0.618 | 177.0 ± 43.7 | 193.8 ± 30.5 | 0.302 |
| RBC (×10^6^/mL) | 5.8 ± 0.2 | 6.5 ± 0.7 | 0.055 | 5.8 ± 0.8 | 5.5 ± 0.6 | 0.385 | 5.8 ± 0.2 | 5.7 ± 1.0 | 0.733 |
| Hct (%) | 27.4 ± 6.4 | 31.8 ± 3.5 | 0.048* | 27.6 ± 5.5 | 26.2 ± 2.7 | 0.141 | 27.4 ± 6.4 | 28.1 ± 2.8 | 0.97 |
| HB (g/dL) | 9.3 ± 1.1 | 10.3 ± 0.9 | 0.035* | 9.27 ± 1.0 | 8.6 ± 0.9 | 0.053 | 9.3 ± 1.1 | 9.2 ± 1.0 | 0.519 |
| MCV (fL) | 49.3 ± 4.1 | 49.2 ± 2.8 | 0.907 | 49.4 ± 3.9 | 48.2 ± 3.6 | 0.671 | 49.3 ± 4.1 | 49.8 ± 3.6 | 0.622 |
| MCH (Pg) | 16.0 ± 1.1 | 16.0 ± 0.9 | 0.953 | 16.1 ± 1.1 | 15.8 ± 0.9 | 0.593 | 16.0 ± 1.1 | 16.2 ± 1.1 | 0.622 |
| MCHC (g/dL) | 32.5 ± 0.9 | 32.6 ± 1.7 | 0.599 | 32.5 ± 0.9 | 32.8 ± 0.9 | 0.326 | 32.5 ± 0.9 | 32.6 ± 0.9 | 0.85 |
| PLT (×10^3^/mL) | 299.1 ± 107.3 | 285.7 ± 106.7 | 0.861 | 291.7 ± 123.6 | 319.8 ± 185.7 | 0.699 | 299.1 ± 107.3 | 273.2 ± 171.3 | 0.569 |
| WBC (×10^3^/mL) | 10.5 ± 3.0 | 13.1 ± 5.3 | 0.318 | 10.3 ± 2.7 | 8.8 ± 2.0 | 0.082 | 10.5 ± 3.0 | 9.8 ± 1.8 | 0.519 |
| Neu (×10^3^/mL) | 3.6 ± 1.2 | 4.0 ± 1.5 | 0.64 | 3.9 ± 1.3 | 3.9 ± 1.6 | 0.868 | 3.6 ± 1.2 | 4.7 ± 1.4 | 0.112 |
| Lym (×10^3^/mL) | 4.6 ± 2.1 | 6.1 ± 3.7 | 0.347 | 4.2 ± 1.9 | 3.4 ± 0.8 | 0.405 | 4.6 ± 2.1 | 3.3 ± 0.4 | 0.381 |
| Mono (×10^3^/mL) | 1.7 ± 1.0 | 2.3 ± 1.2 | 0.215 | 1.5 ± 0.9 | 0.9 ± 0.2 | 0.069 | 1.7 ± 1.0 | 1.0 ± 0.3 | 0.08 |
| Eos (×10^3^/mL) | 0.6 ± 0.4 | 0.7 ± 0.4 | 0.519 | 0.7 ± 0.4 | 0.5 ± 0.3 | 0.122 | 0.6 ± 0.4 | 0.8 ± 0.4 | 0.205 |
| Baso (×10^3^/mL) | 0.1 ± 0.1 | 0.0 ± 0.0 | 0.015* | 0.1 ± 0.1 | 0.1 ± 0.0 | 0.242 | 0.1 ± 0.1 | 0.0 ± 0.0 | 0.08 |

(1) Above-PVL groups: LMS-treated vs. LMS-untreated, (2) LMS-treated groups: BLV-positive vs. BLV-negative, and (3) LMS-treated, BLV-positive groups: Above-PVL vs. Below-PVL. Abbreviations: CBC, Complete blood count; LMS, levamisole; BLV, bovine leukemia virus; TP, total protein; ALB, albumin; GLOB, globulin; T-chol, total cholesterol; RBC, Red blood cell; Hct, hematocrit; HB, hemoglobin; MCV, mean corpuscular volume; MCH, mean corpuscular hemoglobin; MCHC, mean corpuscular hemoglobin concentration; PLT, platelets; WBC, White blood cell; Neu, Neutrophil; Lym, Lymphocytes; Mono, Monocytes; Eosi, Eosinophils; Baso, Basophils. Above-PVL: group with PVL above the cut-off value. Below-PVL: group with PVL below the cut-off value. Values reported are mean ± SD. **P* < 0.05.
