# Supplementary figures and images for: Evaluation of the Therapeutic Effect of Levamisole on Subclinical Mastitis in Bovine Leukemia Virus-Infected Cows Classified by Proviral Load
Source: Animals (Basel). 2025 Jul 21;15(14):2145. doi: 10.3390/ani15142145 (PMC12291948; doi:10.3390/ani15142145)

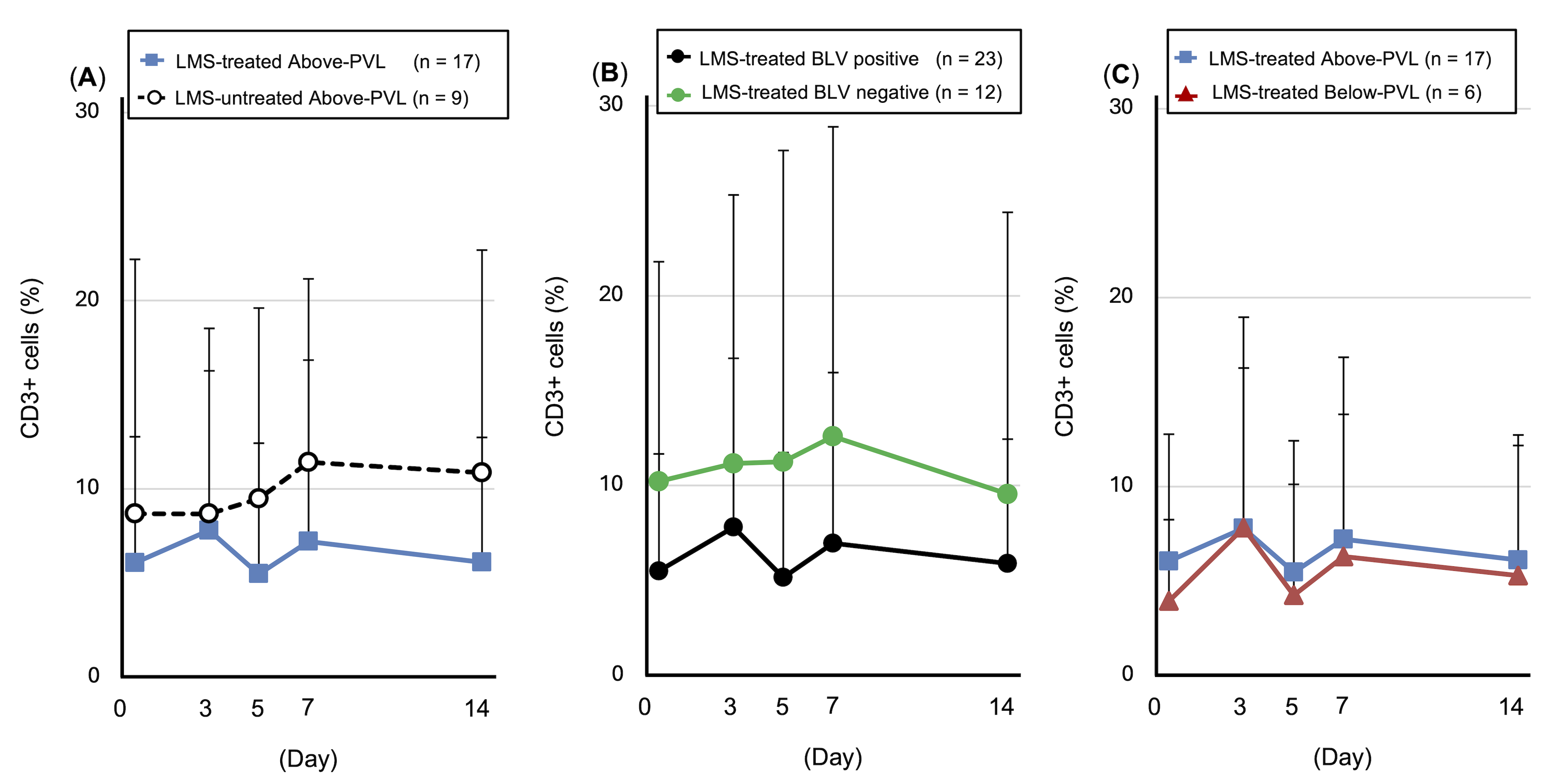

Supplement: Supplementary file 1 [file animals-15-02145-s001.zip › Supplementary Files/Supplementary Figure S6.tiff]

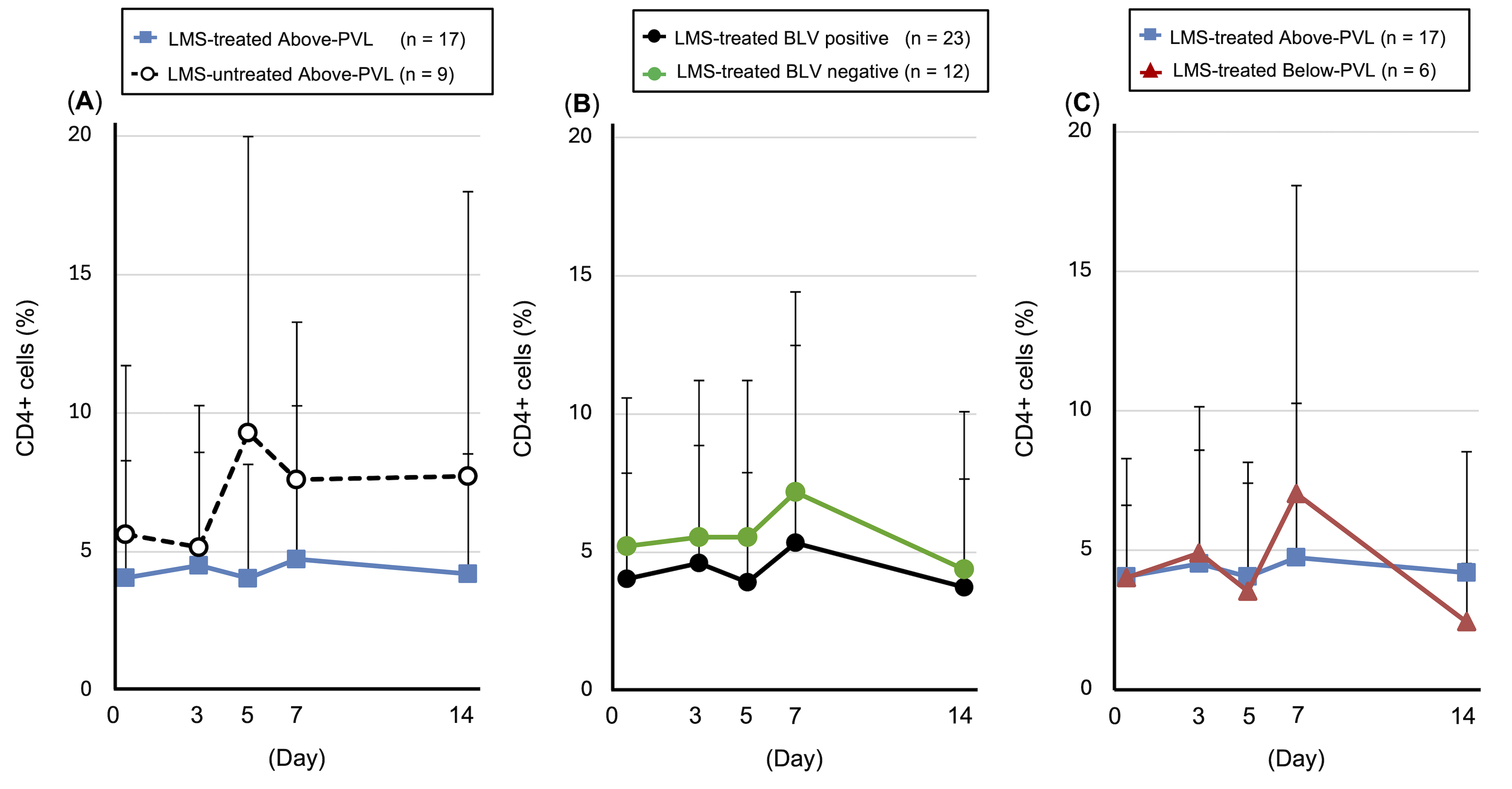

Supplement: Supplementary file 1 [file animals-15-02145-s001.zip › Supplementary Files/Supplementary Figure S7.tiff]

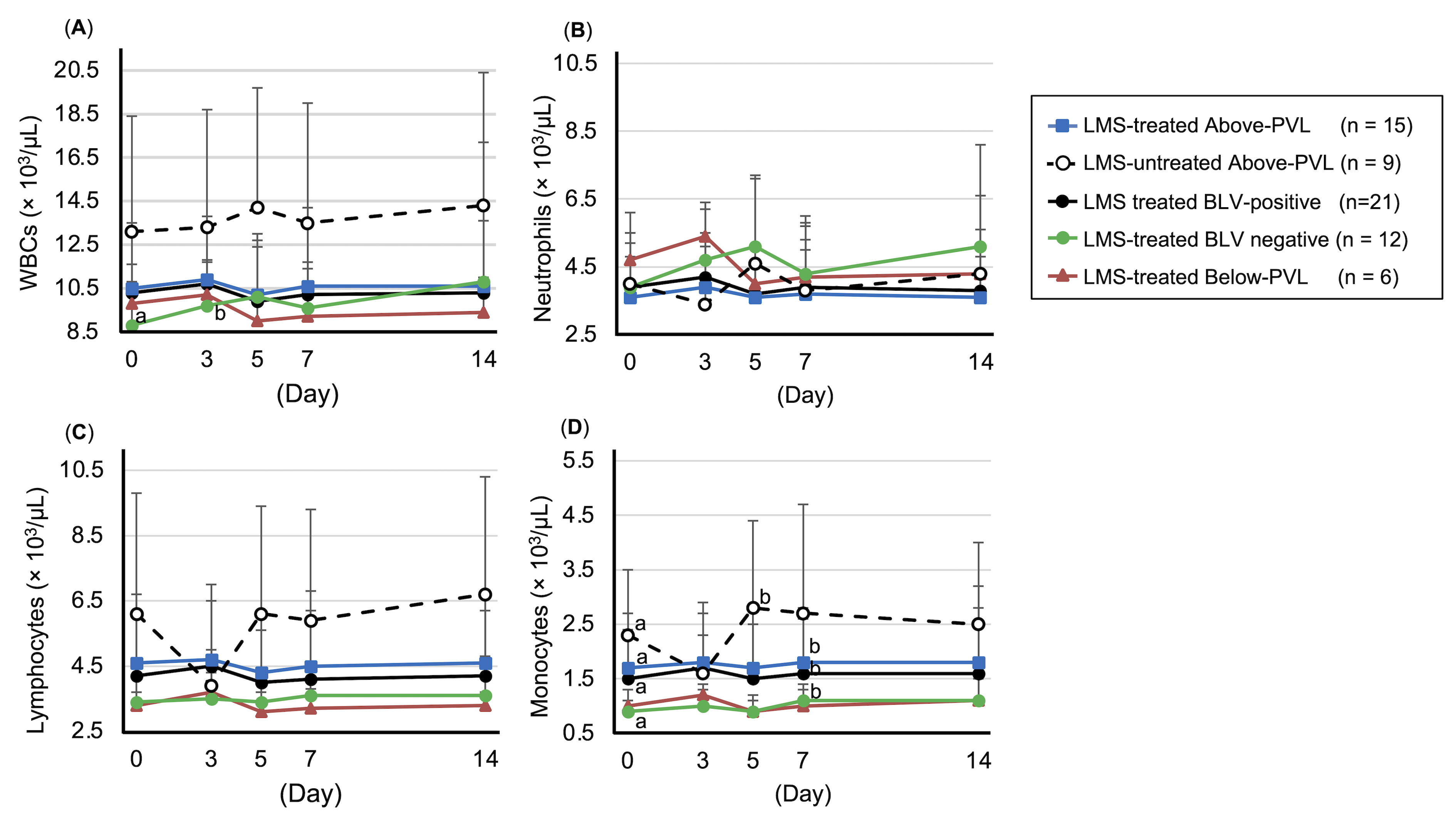

Supplement: Supplementary file 1 [file animals-15-02145-s001.zip › Supplementary Files/Supplementary Figure S1.tiff]

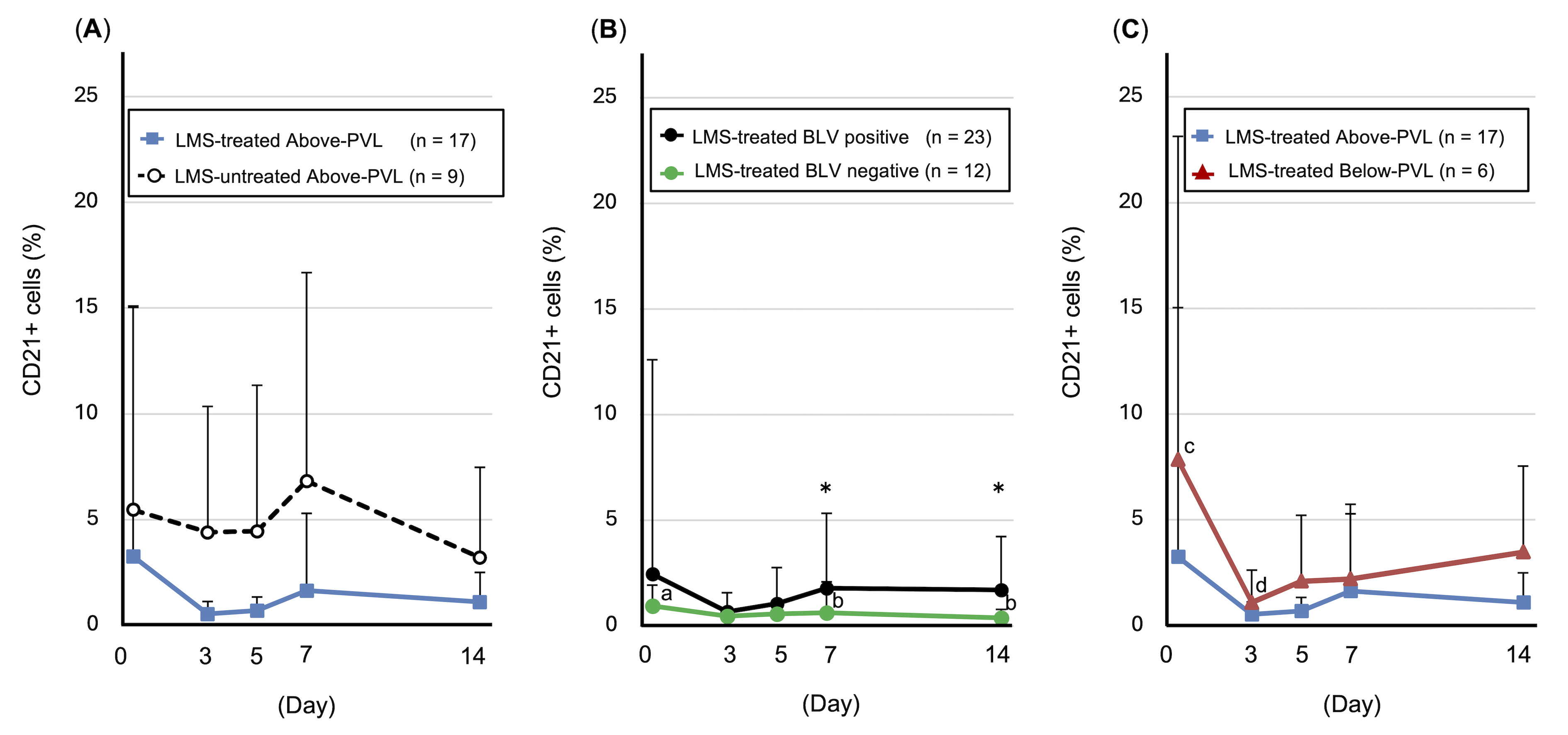

Supplement: Supplementary file 1 [file animals-15-02145-s001.zip › Supplementary Files/Supplementary Figure S2.tiff]

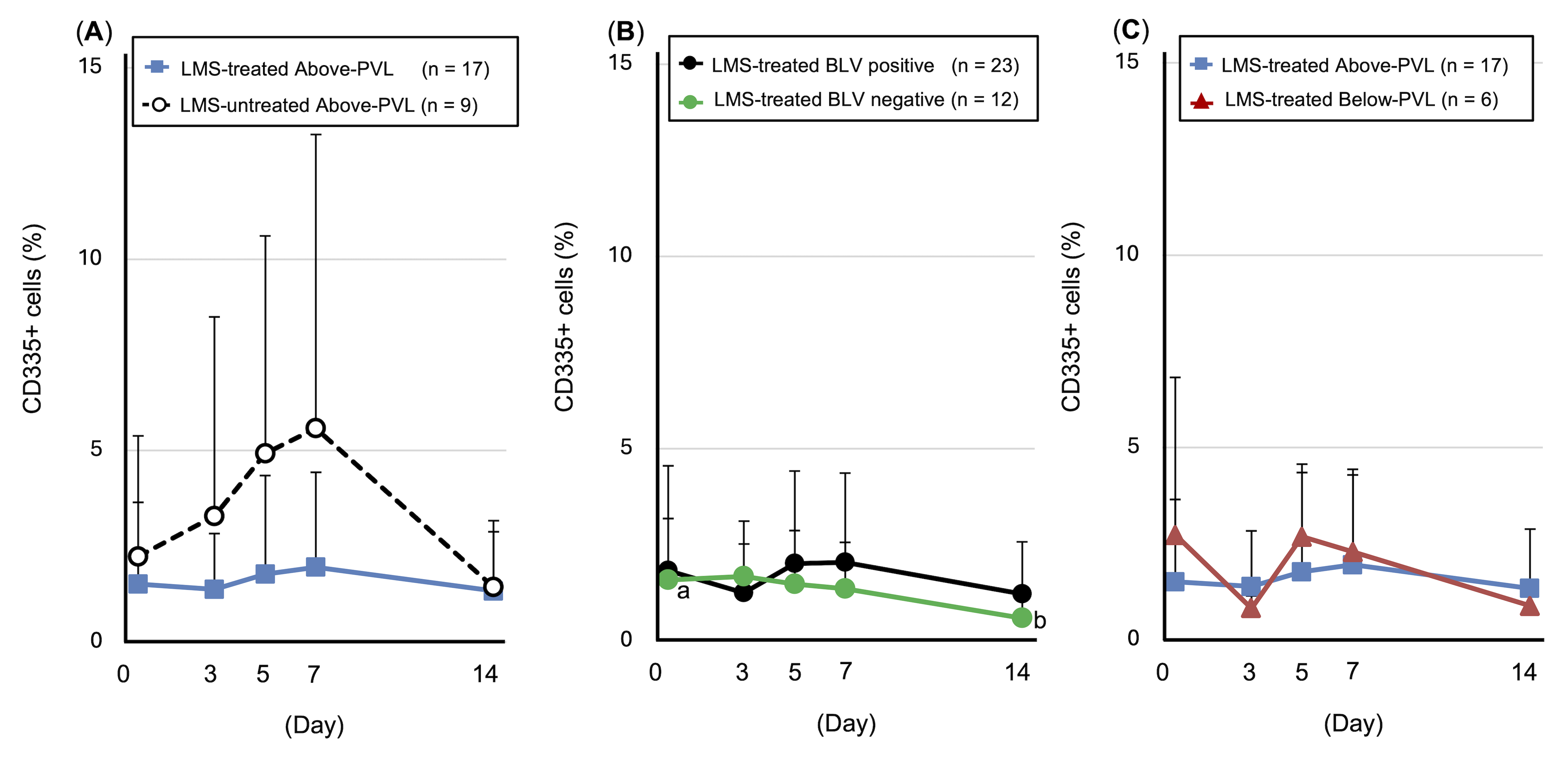

Supplement: Supplementary file 1 [file animals-15-02145-s001.zip › Supplementary Files/Supplementary Figure S3.tiff]

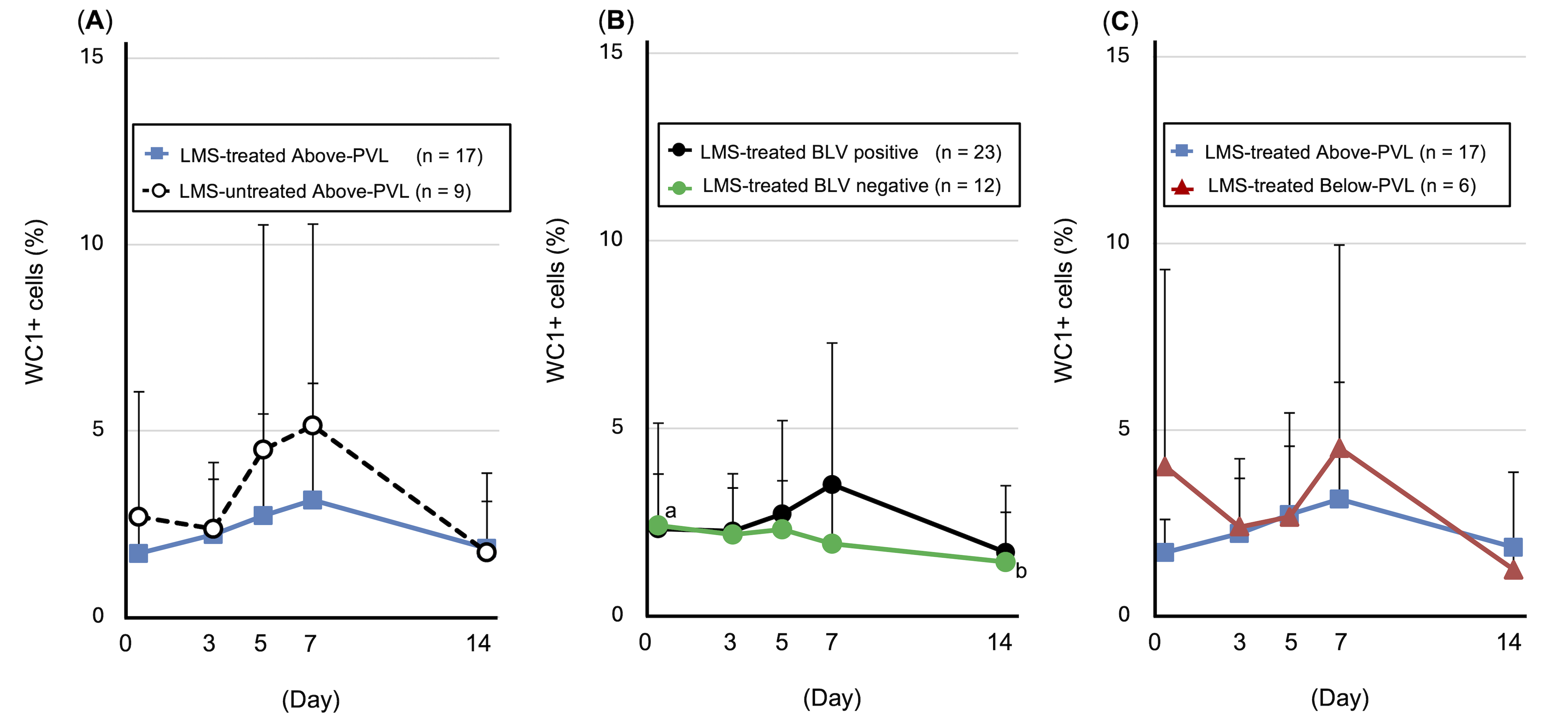

Supplement: Supplementary file 1 [file animals-15-02145-s001.zip › Supplementary Files/Supplementary Figure S4.tiff]

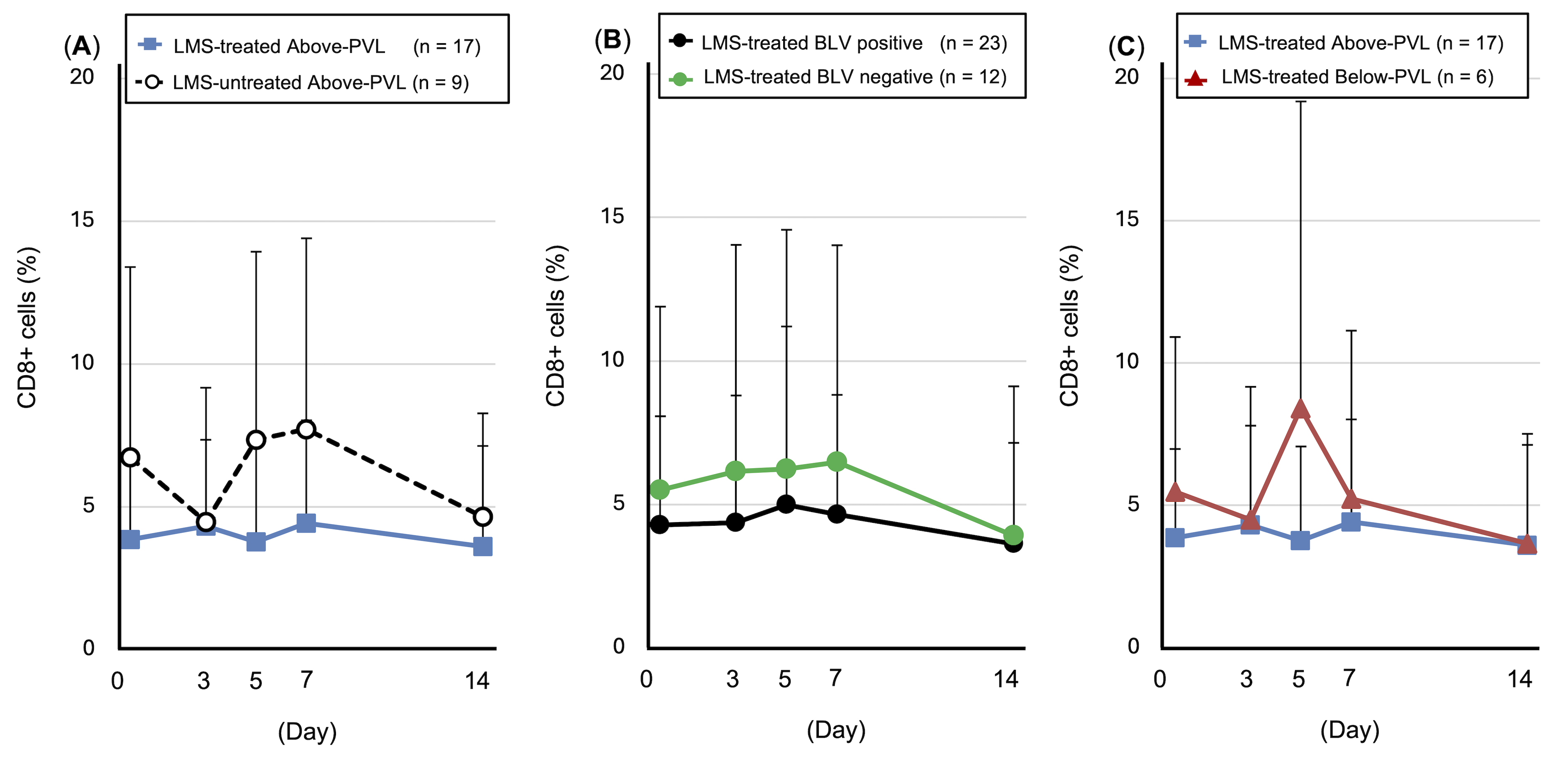

Supplement: Supplementary file 1 [file animals-15-02145-s001.zip › Supplementary Files/Supplementary Figure S8.tiff]

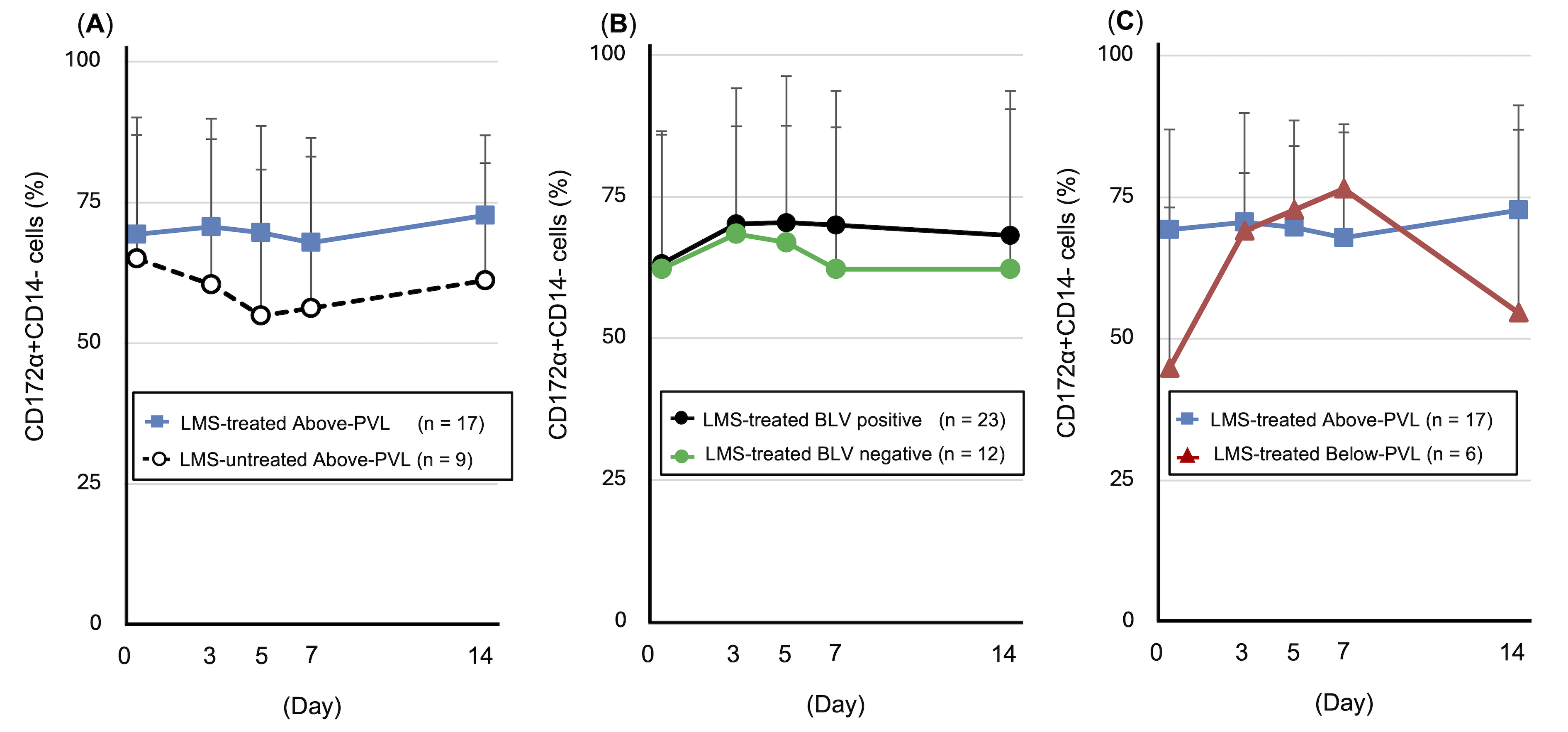

Supplement: Supplementary file 1 [file animals-15-02145-s001.zip › Supplementary Files/Supplementary Figure S5.tiff]
